# Effects of microplastics on reproductive characteristics and mechanisms of the marine rotifer *Brachionus plicatilis*

**DOI:** 10.1038/s41598-024-65047-8

**Published:** 2024-07-02

**Authors:** Taekyoung Seong, Sae Yamamoto, Hisayuki Nakatani, Mitsuharu Yagi, Yusaku Kyozuka, Glenn Satuito, Hee-Jin Kim

**Affiliations:** 1 Graduate School of Integrated Science and Technology, Nagasaki University, 1-14 Bunkyo, Nagasaki, 852-8521 Japan; 2 Co-Creation Management Department, Ryukyu University, 1 Chihara, Nishihara-Cho, Nakagami-Gun, Okinawa Prefecture 903-0213 Japan; 3 Faculty of Fisheries, Nagasaki University, 1-14 Bunkyo, Nagasaki, 852-8521 Japan; 4 Polymeri Materials Laboratory, Chemistry and Materials Program, Nagasaki University, 1-14 Bunkyo, Nagasaki, 852-8521 Japan; 5 Organization for Marine Science and Technology, Nagasaki University, 1-14 Bunkyo, Nagasaki, 852-8521, Japan

**Keywords:** *Brachionus plicatilis*, Rotifer reproduction, Marine microplastics, Natural-aged microplastics, Enzyme activity, Oxidative stress, Ecology, Environmental impact, Marine biology

## Abstract

Microplastic pollution, especially secondary microplastics (MPs), poses a significant threat to marine ecosystems. Despite its prevalence, the impact of natural-aged MPs on marine organisms, hindered by collection challenges, remains poorly understood. This study focused on 1–3 μm natural-aged MPs collected from Japan's coastal sea, investigating their effects on the rotifer *Brachionus plicatilis* sensu stricto and its reproductive mechanisms. Rotifers exposed to varying MP concentrations (0, 20, and 200 particles/mL) over 14-day batch cultures exhibited reduced population growth and fertilization rates. Down-regulation of reproductive genes and up-regulation of oxidative stress-related genes were observed, indicating MP-induced disruptions. Enhanced activities of superoxide dismutase and acetylcholinesterase and elevated malondialdehyde levels further emphasized oxidative stress. These findings underscore the detrimental impact of MPs on rotifer reproductivity, shedding light on the underlying mechanisms.

## Introduction

In the marine environment, plastic wastes undergo weathering processes, ultimately transforming into microplastics (MPs) defined as micro-sized plastic particles due to physicochemical interactions. These MPs generated in the ocean have been found to adsorb persistent organic pollutants (POPs), including polycyclic aromatic hydrocarbons (PAHs), polychlorinated biphenyls (PCBs), polybrominated diphenyl ethers (PBDEs), and dichloro-diphenyl-trichloroethane (DDT)^1–5^. Consequently, MPs have been recognized as potential carriers of these pollutants, thereby enhancing chemical toxicity as accumulation^6–8^. Additionally, the marine MPs have been identified as effective adsorbents of heavy metals. Aged MPs in their natural state possess relatively rough surfaces, leading to a greater capacity for copper adsorption compared to pristine or artificially aged MPs. Consequently, aged MPs exhibit a higher affinity for copper ions^9^. Moreover, MPs have been observed to serve as vectors for heavy metal ions in the marine ecosystem, rapidly adsorbing these elements from nearby sources such as antifouling paints^10^. In the physical realm, MPs in the marine ecosystems exhibit considerable heterogeneity, comprising diverse shapes, sizes, and polymers^11^. The specific shape of MPs has been demonstrated to impact their bioavailability to different species of zooplankton, with each species exhibiting a preference for ingesting a particular shape^12^.

Animal plankton, including rotifers, play a critical role in energy transfer within the aquatic food chain and are capable of transporting pollutants to higher trophic levels through ingestion and subsequent accumulation processes^13–15^. Prior investigations have extensively examined the impact of MPs on animal plankton, specifically regarding various life cycle parameters such as population growth, fecundity, lifespan, reproduction rate, and individual growth^11,15,16^. For instance, in the case of the rotifer species *Brachionus koreanus*, exposure to polystyrene microbeads induced significant physiological alterations that had a pronounced effect on these parameters. Additionally, exposure to the microbeads resulted in the up-regulation of reactive oxygen species (ROS) production and the induction of multiple antioxidant-related enzymes in the experimental plankton^16^. Similarly, negative effects on growth rate, mortality, and reproduction were documented in the freshwater cladoceran *Daphnia magna* upon exposure to nanosized polystyrene beads^17^. In a previous study involving copepods, a decrease in fecundity was measured in test groups exposed to artificial MPs with diameters of 0.5 μm and 6 μm^18^. Furthermore, it has been established that the adverse effects of the MPs on rotifers are contingent upon the size and concentration of the particles^16,18,19^, with life history experiments demonstrating that high concentrations of small-sized MPs (0.07 μm) decrease rotifer survival and reproduction, prolong the time to maturation, and reduce body size at maturation, while large-sized MPs (0.7 and 7 μm) do not significantly affect rotifer life-history traits^19^. Moreover, studies have demonstrated that ingested MPs can disrupt the endocrine system and influence physiological functions, development, and reproduction in aquatic animals^20–22^.

While Vroom et al.^23^ has provided clear evidence of the adverse impacts of MPs on the survival and reproduction of animal plankton, the majority of these studies have utilized artificial primary MPs, such as polystyrene microbeads. There is a scarcity of experiments that utilize MPs found in the marine environment. It has been shown that aged MPs are ingested by a greater number of individuals and at higher rates than pristine plastics in copepod species, indicating the importance of considering aging and weathering effects when investigating the dynamics and effects of plastic ingestion by marine organisms in laboratory settings. Therefore, the present study aims to investigate the effects of marine MPs directly collected from the ocean on the reproduction of marine zooplankton, the euryhaline rotifer *Brachionus plicatilis* as well as elucidate the underlying mechanisms.

## Results

### Rotifer reproduction

**Figure 1:**
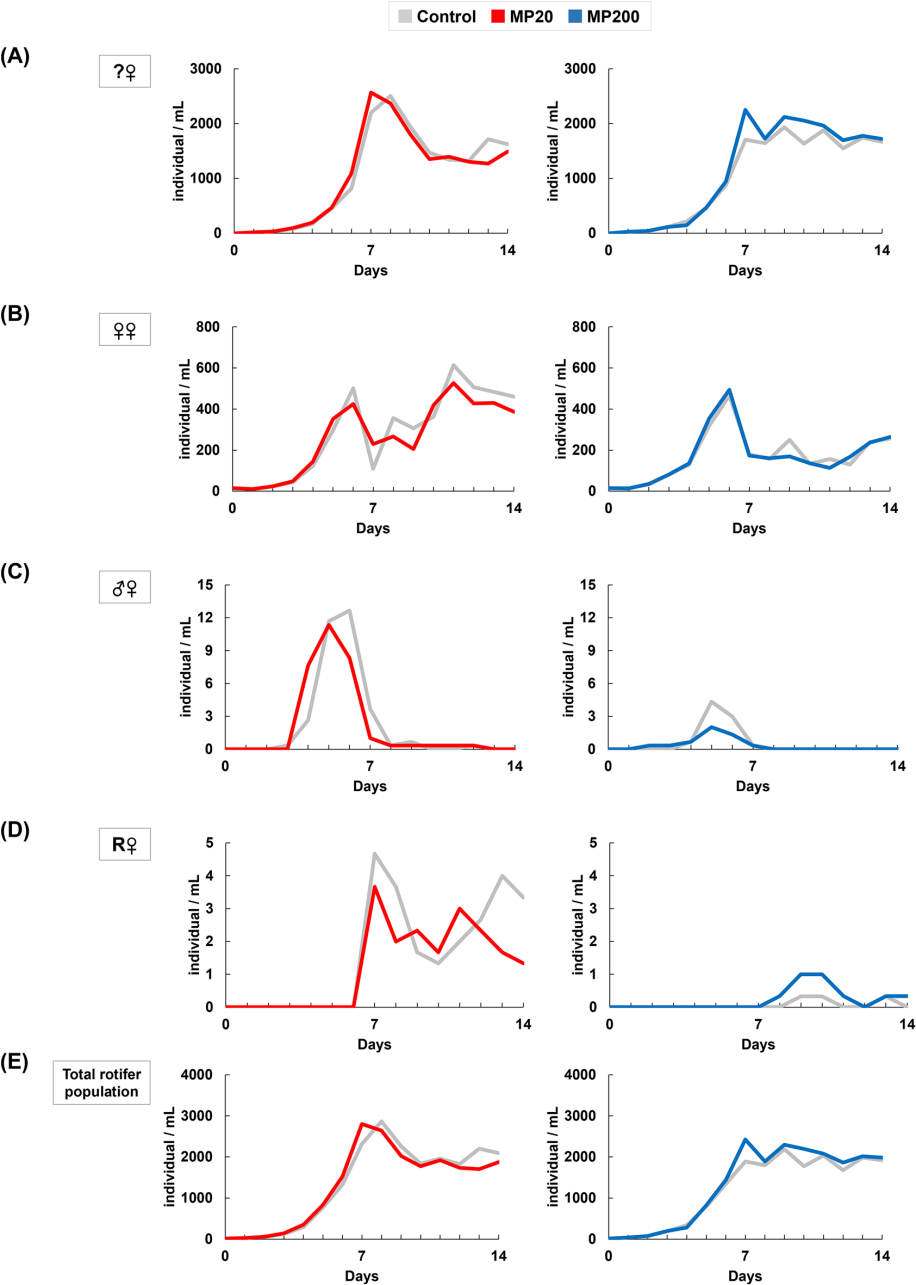
Effects of marine microplastics at different concentrations of 0 particles/mL (control), 20 particles/mL (MP20), 200 particles/mL (MP200) on the population growth of rotifer (individual/mL). Different color lines indicate average population of the rotifer at various reproductive stages: (**A**) female rotifers without eggs (?♀), (**B**) amictic female rotifers carrying female eggs (♀♀), (**C**) mictic female rotifers carrying male eggs (♂♀), (**D**) fertilized mictic females carrying resting eggs (R♀) and (**E**) total population of the rotifer.

No significant differences were observed in the rotifer reproductive parameters, such as population growth rate (*r*), mixis rate, and fertilization rate (Table 1), although, visible variations in population changes were observed between the control group and the experimental groups. The experimental rotifers exposed to MPs exhibited a declining trend in both asexual and sexual reproduction. Specifically, the total population of rotifers exposed to MPs at 20 particles/mL (MP20) showed a decrease during the rearing experiment. Furthermore, the population of female rotifers without eggs (?♀) was lower in the MP20 group compared to the control group. Similarly, the MP20 group displayed a lower growth trend in the population of female rotifers with amictic female eggs (♀♀). Additionally, the population of mictic female rotifers with male eggs (♂♀) exposed to MPs at 200 particles/mL (MP200) showed a decreasing trend compared to the control rotifers. Notably, the number of mictic female rotifers with resting eggs (R♀) in the MP20 group exhibited a decreased pattern compared to the control group (Fig. 1).

**Table 1:** Population growth rate (*r*), mixis rate and fertilization rate of rotifers tested under different microplastic concentrations at 0 particles/mL (control), 20 particles/mL (MP20), 200 particles/mL (MP200).

	Control	MP20	Control	MP200
Population growth rate (*r*)	0.51 ± 0.01	0.51 ± 0.01	0.51 ± 0.01	0.51 ± 0.01
Mixis rate (%)	1.34 ± 0.34	1.37 ± 0.48	0.38 ± 0.17	0.35 ± 0.28
Fertilizaton rate (%)	41.70 ± 9.75	32.12 ± 10.65	8.47 ± 7.50	22.22 ± 23.41

Statistical significance was not observed in the t-test (*p*-value < 0.05, n = 3).

### Relative gene expression

**Figure 2:**
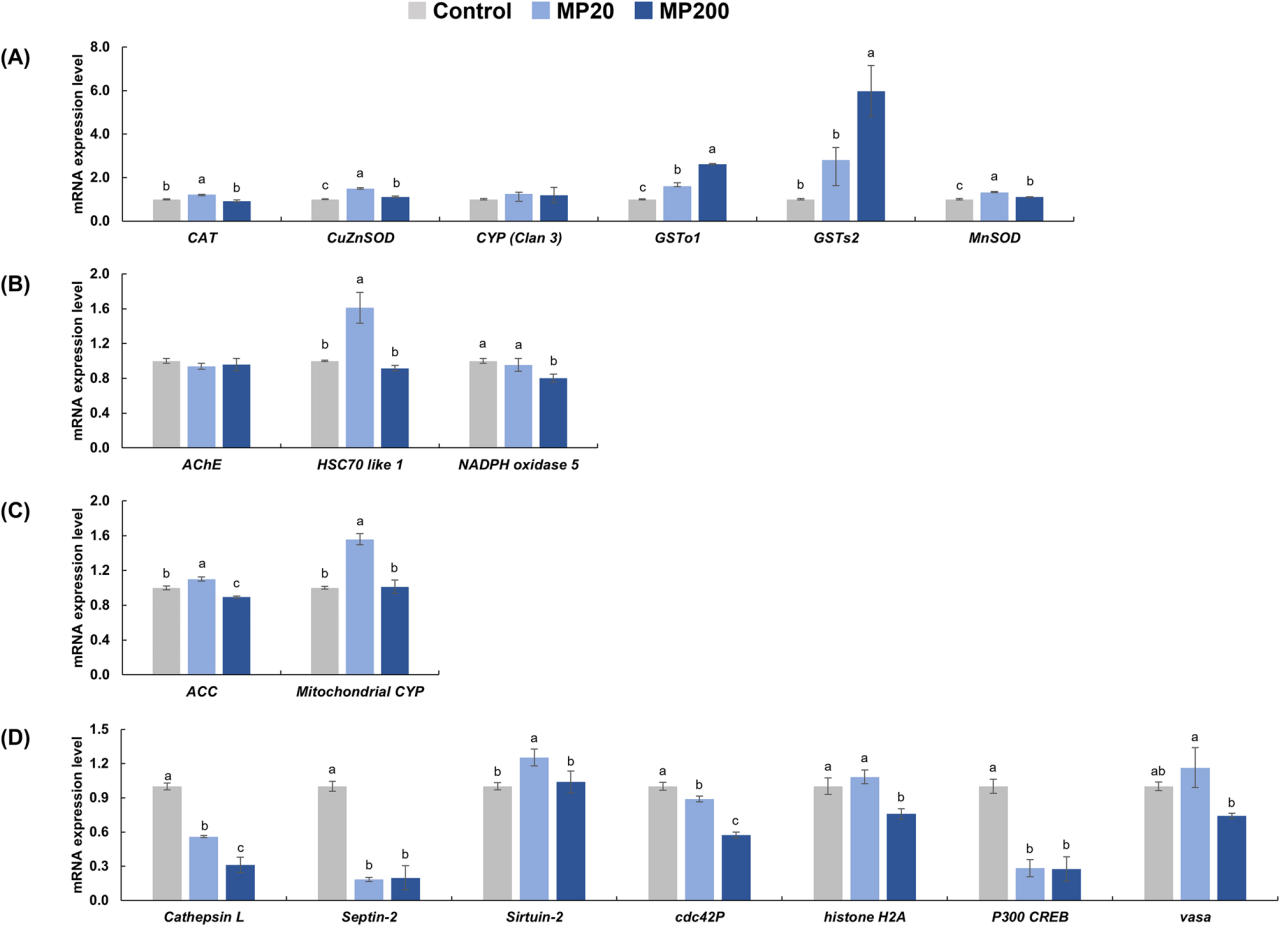
Effects of marine microplastics at different concentrations of 0 particles/mL (control), 20 particles/mL (MP20), 200 particles/mL (MP200) on the relative gene expression of rotifers. The relative gene expression related to antioxidant metabolism (**A**), oxidative stress (**B**), lipid metabolism (**C**), and reproduction (**D**) of the rotifers was analyzed in the present study. Superscript alphabet letters were used to indicate significant differences among the experimental groups (a > b > c, Tukey’s test, *p* < 0.05, n = 3).

Gene expression analysis was conducted to determine the antioxidant metabolism of the experimental rotifer. The expression levels of the following target genes were assessed, and the obtained results were descripted in Fig. 2A: *CAT*, *CuZnSOD*, *CYP (Clan 3)*, *GSTo1* and *GSTs2*, and *MnSOD*. Significant differences in mRNA expression were observed for most of the genes related to antioxidant metabolism, except for *CYP (Clan 3)*. In the MP20 group, there was a significant upregulation in gene expression for *CAT*, *CuZnSOD*, *GSTo1*, *GSTs2*, and *MnSOD*. Specifically, *CAT* gene expression was upregulated by 1.2-fold, *CuZnSOD* by 1.5-fold, *GSTo1* by 1.6-fold, *GSTs2* by 2.8-fold, and *MnSOD* by 1.3-fold. In the MP200 group, there was a significant upregulation in gene expression for *CuZnSOD*, *GSTo1*, *GSTs2*, and *MnSOD*. Nevertheless, several gene expressions were upregulated such as *CuZnSOD* by 1.1-fold, *GSTo1* by 2.6-fold, *GSTs2* by 6.0-fold, and *MnSOD* by 1.1-fold.

To assess oxidative stress in the experimental rotifer, we examined the gene expression levels of *AChE*, *HSC70 like 1*, and *NADPH oxidase 5* and the results were represented in Fig. 2B. In the MP20 group, there was a significant increase in gene expression of *HSC70 like 1*, which was upregulated by 1.6-fold. On the other hand, the MP200 group showed a significant decrease in the gene expression of *NADPH oxidase 5*, which was downregulated by 0.8-fold.

The lipid metabolism of the experimental rotifer was assessed by examining the gene expression levels of *ACC* and *mitochondrial CYP* what results were confirmed in Fig. 2C. In the MP20 group, there was a significant increase in gene expression for both *ACC* and *mitochondrial CYP* compared to the control group. *ACC* gene expression was upregulated by 1.1-fold, while *mitochondrial CYP* gene expression was upregulated by 1.6-fold. In contrast, in the MP200 group, there was a significant decrease in gene expression of *ACC* compared to the control group, with a downregulation of 0.9-fold.

Figure 2D illustrates the gene expression patterns associated with asexual and sexual reproduction in response to different concentrations of MPs. The reproductive behavior of the experimental rotifers was assessed by examining the gene expression levels of several genes associated with asexual reproduction, including *Cathepsin L*, *Septin-2*, and *Sirtuin-2*, as well as those associated with sexual reproduction, such as *cdc42P*, *histone H2A*, *P300 CREB*, and *vasa*. In the MP20 group, the expression of the *Cathepsin L* gene was downregulated by 0.6-fold and *Septin-2* by 0.2-fold, both of which are related to asexual reproduction. Additionally, the gene expression of *cdc42P* was downregulated by 0.9-fold and *P300 CREB* by 0.3-fold, both related to sexual reproduction. However, the expression of *Sirtuin-2*, which is involved in both asexual and sexual reproduction, was significantly upregulated by 1.25-fold compared to the control group. In the MP200 group, the expression of *Cathepsin L* was downregulated by 0.3-fold, *Septin-2* by 0.2-fold, *cdc42P* by 0.6-fold, *histone H2A* by 0.8-fold, and *P300 CREB* by 0.3-fold.

### Generated ROS levels

**Figure 3:**
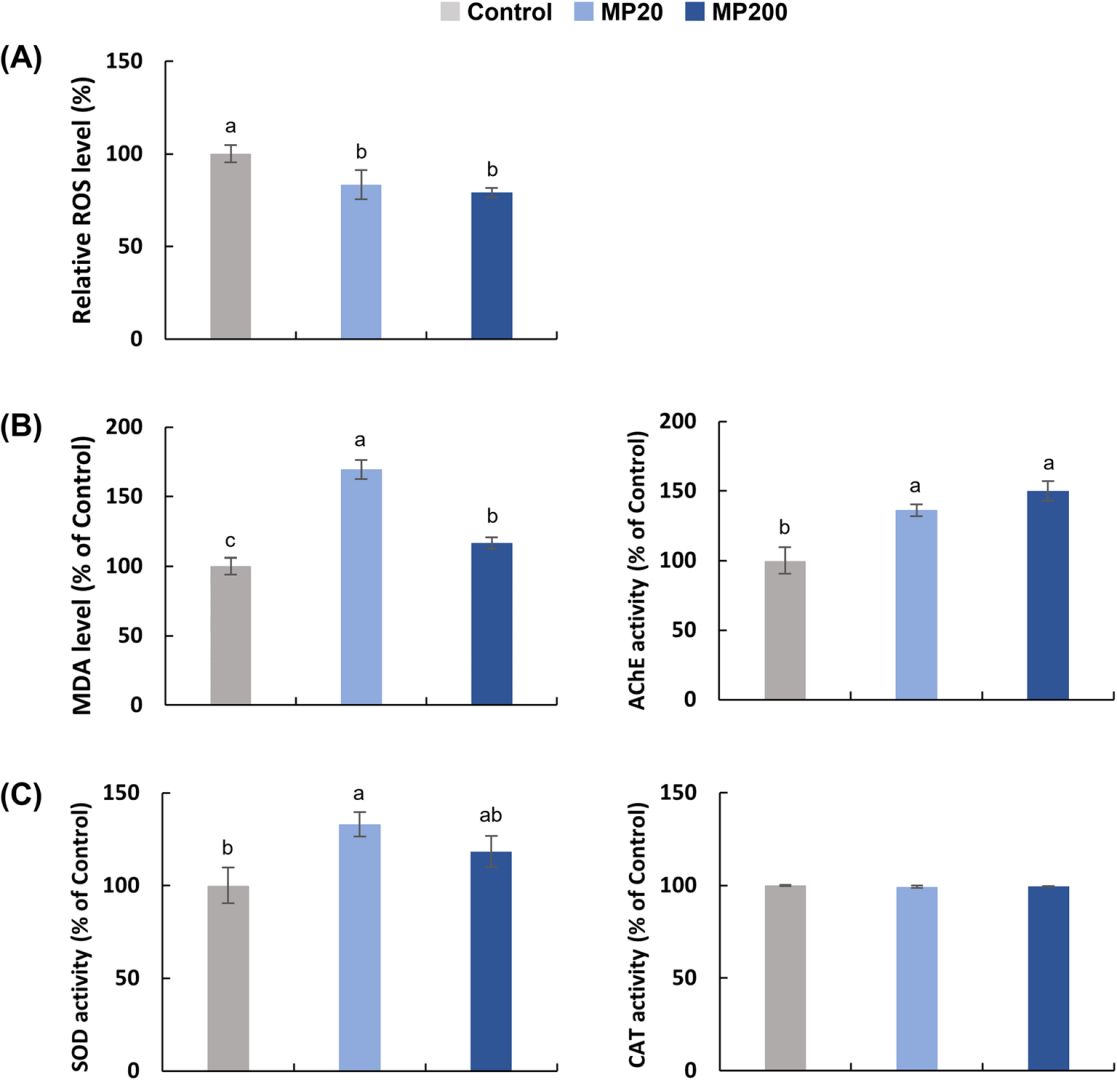
Effects of marine microplastics at different concentrations of 0 particles/mL (control), 20 particles/mL (MP20), 200 particles/mL (MP200) on the intracellular ROS levels and enzymatic activities. Three aspects were assessed: (**A**) intracellular ROS levels, (**B**) enzymatic activity associated with oxidative stress, and (**C**) antioxidant responses in the rotifer tested. Superscript alphabet letters were used to indicate significant differences among the experimental groups (a > b > c, Tukey's test, *p* < 0.05, n = 3).

After 6 days of exposure, the intracellular levels of ROS were found to be significantly decreased (*p* < 0.05) in all the MP groups tested, as shown in Fig. 3A. The experimental rotifers exhibited ROS levels that were approximately 83.5%, and 79.1% of its level in the control group.

### Relative MDA levels and enzymatic activities (AChE, SOD, and CAT)

The relative levels of MDA and enzymatic activities associated with oxidative stress and antioxidant responses in experimental rotifers exposed to different concentrations of MPs revealed significant differences (*p* < 0.05), except for relative CAT activity.

Regarding oxidative stress biomarkers, the MDA level showed a significant upregulation in the MPs groups, reaching 169.4% in the MP20 group and 116.5% in the MP200 group (Fig. 3B). Furthermore, the relative activity of AChE was significantly upregulated in the MPs groups, measuring 136.1% in the MP20 group and 150.0% in the MP200 group.

Regarding antioxidant response biomarkers, the activity of SOD was significantly upregulated in the MPs groups, reaching 133.2% in the MP20 group and 118.4% in the MP200 group (Fig. 3C).

## Discussion

Monogonont rotifers reproduce through cyclical parthenogenesis, which involves frequent asexual reproduction and sporadic sexual reprodcution^14,24–31^. The transition from asexual (amictic) to sexual (mictic) reproduction is influenced by a range of internal and external factors, including genotype, diet, population density, photoperiod, temperature, and salinity^29^. Notably, the reproductive physiology of amictic and mictic females varies significantly. Additionally, their sizes and the types of eggs they produce are distinct from one another^27–29^. Furthermore, their mechanisms for oogenesis differ, as amictic females produce diploid eggs while mictic females produce haploid eggs^24^. Consequently, these distinctions in reproductive physiology lead to differential responses to various environmental conditions in amictic and mictic females^19,29,30^. In this experiment, no statistically significant differences were observed in the reproductive parameters of rotifers tested, while there was a noticeable decline in the population density of female rotifers with amictic female eggs (♀♀), mictic female rotifers with male eggs (♂♀), and the number of mictic female rotifers with resting eggs (R♀). These findings indicate that both sexual and asexual reproductions were influenced by the presence of marine MPs.

Analysis of relative gene expression revealed significant downregulation of genes associated with asexual reproduction, such as *Cathepsin L* and *Septin-2*, which play roles in asexual female development and yolk processing^24,32,33^. Additionally, genes related to sexual reproduction, including *cdc42P*, *histone H2A*, and *P300 CREB*, which are involved in gametogenesis and chromosome segregation, were also significantly downregulated^32,34,35^. Furthermore, the conserved germ cell-specific gene *vasa*, responsible for germ cell formation, and crucial for gametogenesis and embryogenesis, showed decreased expression in the MP200 group compared to the control group^36–38^. Conversely, the aging-related gene *Sirtuin-2*, known to be associated with slowing aging in mammals, exhibited upregulation in the MP20 group^39–41^. Even if this study could not observe aging patterns of rotifers related to MP exposure through individual cultures, the difference may exist to lead maximum population density.

Previous studies have established a link between oxidative stress and sperm abnormalities, highlighting the adverse impact of elevated oxidative stress levels on mitochondrial membrane potential, electron transport chain-related male fertility, and sperm quality^42–44^. In the context of our experiment, the observed reduction in the prevalence of reproductive patterns in rotifers, such as female rotifers bearing amictic female eggs (♀♀), mictic female rotifers producing male eggs (♂♀), and mictic female rotifers forming resting eggs (R♀), is likely attributed to elevated oxidative stress levels and the consequent accumulation of oxidative stress.

ROS can be generated in the process of normal metabolism, while heightened in response to toxic substances and environmental stress, leading to detrimental effects on organismal growth^21,45^. Excessive ROS production results in oxidative stress, causing cellular dysfunction and mortality^46,47^. It has been known that MPs induce oxidative stress and disrupt antioxidant systems in organisms^16^. Therefore, assessing the antioxidant response is crucial for elucidating the toxic mechanisms by which MPs exerts its harmful effects on rotifers^48^. In our experiment, rotifers exposed to marine MPs exhibited lower relative ROS levels compared to the control group, contrary to our expectations. However, biomarkers indicating damage caused by ROS, such as relative MDA levels, relative AChE, and SOD activity, were significantly higher in the MP-exposed group compared to the control group. Also, in the analysis of relative gene expression, the gene *HSC70 like1*, associated with oxidative stress, was significantly upregulated in the control group, and all genes related to antioxidant metabolism (*CAT*, *CuZnSOD*, *CYP (Clan 3)*, *GSTo1*, *GSTs2*, and *MnSOD*) were upregulated regardless of MP concentration. Upon analyzing these patterns, the diminished levels of ROS in rotifers from the marine MPs group, in contrast to the control group, can be ascribed to two plausible explanations. It is conceivable that rotifers actively synthesized enzymes, such as SOD, which are involved in the mitigation of reactive oxygen species, thereby accounting for the observed trend. Alternatively, this distinction could stem from a diminishment in metabolic activity within the rotifers. These findings serve as compelling evidence that rotifers exposed to marine MPs underwent oxidative stress.

Exposure of aquatic animals to contaminants, including MPs, not only increases ROS production but also triggers the subsequent activation of antioxidant mechanisms^49–51^. It has been known that the extent of oxidative damage depends on the effectiveness of an organism's antioxidant defense system^52^, including SOD, CAT, CYP, glutathione (GSH), and glutathione peroxidase (GPx), effectively eliminates ROS^53–55^. Previous studies investigating the impact of MPs exposure on organisms have presented evidence that heightened activities of SOD and CAT effectively alleviate the harmful consequences of ROS^56,57^. In the present study, the observed reduction in ROS levels in the MP-exposed groups can be attributed to the enhanced enzymatic activity of SOD, a key component of the antioxidant metabolism.

## Conclusion

Our experiment provides valuable insights into the impact of marine MP exposure on organisms, particularly rotifers. The findings reveal that marine MPs induce oxidative stress and disrupt the antioxidant defense systems in rotifers, as evidenced by the significant alterations in biomarkers associated with ROS and antioxidant activity. Despite the unexpected lower relative ROS levels in MP-exposed rotifers compared to the control group, biomarkers indicating oxidative damage were significantly higher in the MP-exposed group. These results suggest that rotifers exposed to marine MPs experience oxidative stress, potentially impairing their reproductive patterns and overall fitness. Further investigations are necessary to elucidate the specific mechanisms by which MPs induce oxidative stress and impair reproductive processes in rotifers. In this study, a comparison between marine MPs and artificial MP beads was not conducted. However, future investigations should include such a comparison to examine the actual toxicity of marine MPs on marine organisms. As MP pollution continues to be a global concern, further research in this area is vital for a comprehensive understanding of its ecological implications and potential threats to aquatic ecosystems.

## Materials and methods

### Marine microplastics

**Figure 4:**
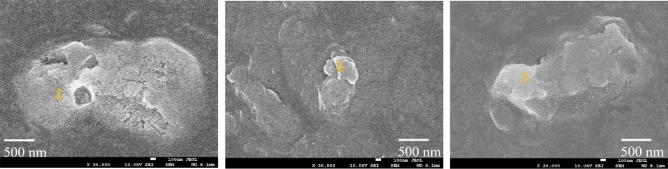
Microscopic view of microplastic particles collected from coastal sea of Kyushu area, illustrating the diversity in size and shape. Scale bar = 500 nm.

To investigate the impact of marine MPs on rotifer reproductivity and elucidate the underlying molecular mechanisms, we conducted a comprehensive sampling effort of marine MPs in the coastal sea of the Kyushu area, Japan (32°46.49′N129°43.80′E). Employing a subsequent filtration with mixed cellulose ester membrane filter (Membrane filter, pore size 1.00 μm and 3.00 μm, Advantec MFS Inc., Japan) enabled us to obtain a purified subset of MPs, specifically targeting particles approximately 3 μm (1–3 μm of size distribution) in diameter. The size of marine MPs were chosen to match the size of the feed phytoplankton (2 to 4 μm^58^), which could potentially have a greater impact on the experimental rotifers through their digestive system, as they are filter feeders. To determine the accurate size distribution of marine MPs filtered, we measured the size of randomly selected 30 particles with an inverted microscope (ECLIPSE Ts2, Nikon Instruments Inc., New York, USA) (Fig. 4).

### Target species

We employed the marine rotifer *B. plicatilis* sensu stricto (NH1L strain) with an average lorica length of 275 μm^59^. The rotifers used in this study were derived from populations of *B. plicatilis* (NH17L strain) that have been maintained at the Aquaculture Biology Laboratory, Nagasaki University, Japan, for over two decades. During the experiment, the rotifers were maintained in sterilized seawater at a salinity of 22 parts per thousand (ppt) and a water temperature of 25 °C in a controlled incubator (MIR-254S-PJ, PHCbi, Tokyo, Japan) under complete darkness. The culture medium was prepared by diluting natural seawater with distilled water (RFP841AA Pure Water Purifier, Adventec Manufacturing Inc., Ontario, Canada), followed by filtration (0.45 μm Membrane filter, Nihon Millipore K.K., Tokyo, Japan) to remove small particles and autoclaving (at 121 °C for 20 min). Additionally, the rotifers were fed on cultivated microalgae cells of *Nannochloropsis oculata* ad libitum.

### Population growth of the rotifer

To assess the impact of marine MPs on rotifer reproductivity, we initiated batch cultures of rotifers which were maintained at 22 ppt and 25 °C with feeding of *N. oculata* at 7 × 10^6^ cells/mL. From the one of these cultures, female rotifers carrying amictic eggs were pipetted out and inoculated into a prepared 15 mL glass bottle at a density of 1 individual per 1 mL. The experimental rotifers were subjected to specific concentrations of marine MPs. A concentration of 20 particles/mL, denoted as MP20, was selected to represent a realistic high level of MPs in marine environments^60,61^, thereby facilitating an assessment of the current impact of marine microplastics on local ecosystems. Additionally, a concentration of 200 particles/mL, denoted as MP200, was employed to assess potential future effects arising from the ongoing and escalating accumulation of plastic waste in marine ecosystems. These concentrations were maintained over a 14-day period during the experimental exposure of rotifers to marine MPs. The culture experiment with rotifers was carried out with three replicate trials. Throughout the culture period, we daily counted the total number of rotifers and female rotifers at various reproductive stages, including female rotifers without eggs (?♀), amictic female rotifers carrying female eggs (♀♀), mictic female rotifers carrying male eggs (♂♀), and fertilized mictic females carrying resting eggs (R♀). We estimated the effects of MPs on sexual and asexual reproductivity by calculating population growth rate, mixis rate, and fertilization rate using the following equations.

$$\text{Population growth rate } \left(r\right)=\frac{\text{ln}{N}_{t}-\text{ln}{N}_{0}}{t}$$

In the equation for population growth (*r*), *t* represents the culture day, *N*_*t*_ and *N*_*0*_ denote the total number of female rotifers on day *t*, and *N*_0_, respectively.

### Rotifer incubation for the assessment of associated biomarkers

To investigate the impact of marine MPs on rotifers, we conducted an incubation experiment using the same rotifer strain and experimental conditions employed in the population growth study. The rotifers were exposed to three different concentrations of marine MPs: 0 (control), 20, and 200 particles/mL, over a 6-day period. This incubation was carried out in 300 mL bottles, each containing a density of 10 rotifers per mL, and the experiment was conducted for 6 days, replicated in triplicate. This experimental setup was meticulously designed to ensure a sufficient population of rotifers for subsequent assessments. These assessments included analyzing gene expression associated with oxidative stress, encompassing the generation of reactive oxygen species (ROS), antioxidant metabolism, and reproduction, which were all analyzed using RT-qPCR. It also allowed for the measurement of ROS levels and the evaluation of the relative activities of key enzymes engaged in antioxidant defense, specifically superoxide dismutase (SOD), catalase (CAT), and acetylcholinesterase (AChE). Additionally, it facilitated the quantification of the relative amount of malondialdehyde (MDA).

### Relative gene expression

For RNA extraction, approximately 6000 rotifers were sampled and NucleoSpin® RNA kit (Takara Bio Inc, Shiga, Japan) was used following the manufacturer's instructions. The extracted RNA underwent treatment with a TURBO DNA-free™ kit (Thermo Fisher Scientific Inc., Waltham, Massachusetts, USA) to eliminate genomic DNA contamination. The quality and quantity of RNA were assessed spectrophotometrically at 230 nm, 260 nm, and 280 nm using a NanoDrop 2000 spectrophotometer (Thermo Fisher Scientific, Waltham, Massachusetts, USA). Subsequently, complementary DNA (cDNA) synthesis was carried out utilizing the PrimeScript™ II 1st strand cDNA Synthesis Kit (Takara Bio Inc, Shiga, Japan), and the resulting cDNA was stored at − 20 °C until further analysis.

Reverse transcription quantitative polymerase chain reaction (RT-qPCR) was performed using 1 μg of cDNA template, 0.5 μL of forward and reverse primers (100 μM), and 10 μL of TB Green Premix Ex Taq (2 ×) (Takara Bio Inc, Shiga, Japan), making a total volume of 20 μL. The RT-qPCR assay was carried out on a QuantStudio 1 Real-Time PCR System (Thermo Fisher Scientific, Waltham, Massachusetts, USA) with the following thermal cycling conditions: an initial denaturation at 94 °C for 4 min, followed by 43 cycles of denaturation at 94 °C for 30 s, annealing at 55 °C for 30 s, and extension at 72 °C for 30 s. Melting curve analysis was performed to confirm the amplification of specific products, involving cycles at 95 °C for 10 s, 55 °C for 1 min, and 80 cycles at 55 °C for 10 s, with a 0.5 °C increment per cycle^62^. The genes subjected to analysis can be located in Table 2, while their corresponding primer sequences were designed based on several previous studies^62–66^. The *elongation factor 1-α* (*EF1-α*) gene was selected as a reference due to its stable expression across and within the experimental groups. The transcriptional levels were calculated using the 2^(−ΔΔCt)^ method^67^. All experiments were conducted in triplicate to ensure reliability and reproducibility of the results.

**Table 2:** GenBank accession numbers and primer sets employed in this study for assessing the associated biomarkers associated with oxidative/antioxidant activity, lipid metabolism, and rotifer reproduction.

	GenBank accession no.	Gene	Primer sequence (5′ to 3′)	References
Antioxidant metabolism	BAH28837	*Catalase* (CAT)	F: GGAATCGAGCCATCACCAGA	Han et al.^64^
			R: GCATTGTGGACCATCACGTT	
	RNA21457	*Manganese suproxide dismutase* (*MnSOD*)	F: AGCACCAACTGGAACTTTGA	
			R: CTGATGGCCAGAGATCCGTC	
	Bp_167200T	*Copper/zinc superoxide dismutase* (*CuZnSOD*)	F: AAGCAGTGGCAGTCCTAATTGG	Han et al.^62^
			R: CGCCGAACTGGTGAATGTGA	
	Bp_CYP3045A2	*CYP* (*Clan3*)	F: GCTGACTATGGACTCGCTTTG	Park et al.^65^
			R: CAGATCGGGTCCAACTCG	
		*Glutathione S transferase (GSTs2)*	F: TGCAAGAGGTCGAGCTGAG	
			R: TGACCCAGAGGAGCTTTCTT	
	BRGM023370T0	*Glutathione S transferase (GSTo1)*	F: TTGCCGAAGACTTGCTCAAA	
			R: CCAATCTCTCGATCCACGGC	
Oxidative stress	Bp_105060T	*Acetylcholinesterase (AChE)*	F: TCCATCTGAGGCAGGTAGGT	Han et al.^63^
			R: TTCGCACGCATTCAATTCCA	
	AB076052.1	*heat shock protein 70 (HSC70 like 1)*	F: CGACAACCGACTGGTCAACC	Han et al.^62^
			R: GCTCTGGTGATGCTCGTGTAG	
	Bp_056850T	*NADPH oxidase 5*	F: CATCGCCTCTTCGCCTACTTG	
			R: CACCATCGCTGTCCACATCAA	
Lipid metabolic	Bp_011640T	*Acetyl-CoA carboxylase* (*ACC*)	F: GCATTCAGCACACGCAAGA	Han et al.^62^
			R: GAGGAGGAAGAGGAGCAGTC	
	Bp_CYP3335A1	Mitochondrial *CYP*	F: GATCCTGAAATTGCAGAACAAG	
			R: GTTCTTAAAATTCGCCAACG	
Rotifer reproduction	Bp_082650T	*Cathepsin L*	F: CAAGTTCAATGGCACGCTCAG	Han et al. ^62^
			R: CAGCATCCACCGCAGTCAA	
	Bp_102670T	*Septin-2*	F: TGCGGTAGTTGGTTCTAGTCAG	
			R: GGAGCCTAGCCGTTCAGATC	
	Bp_195460T	*Sirtuin-2*	F: GCGAATGCTTGCCAACTGAATT	
			R: CAGCCAGCGGTAGCCTAGAA	
	Bp_184930T	*cdc42P*	F: TGTAGTAGGAGACGGAGCAGTG	
			R: AGACGGTCGTAGTCCTCTTGG	
	Bp_042560T	*histone H2A*	F: ATCGGCTGTCCTGGAATACTTG	
			R: ACACCACCTTGAGCAATAGTGA	
	Bp_148890T	*P300/CREB-binding protein (P300 CREB)*	F: TGCTCATATTCGGCTCACACTT	
			R: TGTCATTGGAACCAGTGGCTAA	
	GU969245.1	*vasa*	F: GAGTCAGTTGAGCGGCATGC	Zhang et al.^66^
			R: AGCCGAAGAAAACACCACCG	
Reference gene	AB513493.1	*Elongation factor 1-alpha 1 ((EF1-α)*	F: GACGCCATTGTTCCACCATCA	Zhang et al.^66^
			R: GGCTGGAGCAAAAGTGACAAC	

### Investigation of reactive oxygen species levels and enzymatic/biomarker activities

In addition to gene expression analysis, we also measured ROS levels and assessed the relative activities of key enzymes involved in antioxidant defense, namely superoxide dismutase (SOD), catalase (CAT), and acetylcholinesterase (AChE). Furthermore, we quantified the relative amount of malondialdehyde (MDA), which serves as a biomarker of lipid peroxidation and oxidative damage. These measurements allowed us to evaluate the oxidative stress status and the potential impact of MPs on enzymatic and biomarker activities in rotifers.

The levels of ROS were determined in rotifers exposed to marine MPs using a fluorogenic probe called 2′,7′-dichlorofluorescein diacetate (H2DCFDA) from Sigma-Aldrich. This probe undergoes oxidation by ROS, resulting in the production of fluorescent 2′,7′-dichlorofluorescein (DCF). The fluorescence intensity was measured using a Cytation™ 3 multimode plate reader (BioTek Instruments, Winooski, Vermont, USA) with excitation and emission wavelengths set at 485 nm and 520 nm, respectively. To prepare the samples, approximately 5000 rotifers were homogenized in a buffer solution containing 0.32 M sucrose, 20 mM 4-(2-hydroxyethyl)-1-piperazineethanesulfonic acid (HEPES), 1 mM MgCl_2_, and 0.5 mM phenylmethylsulfonyl fluoride (PMSF) (pH 7.4, adjusted with NaOH and HCl) using a Branson Sonifier 150 (Eerson Electiric Co., St. Louis, Missouri, USA). The homogenate was then centrifuged at 10,000×*g* for 20 min at 4 °C, and the resulting supernatant was transferred to new microtubes for further analysis. The reaction mixture consisted of 170 μL of phosphate-buffered saline, 20 μL of H2DCFDA probe, and 10 μL of the prepared sample. The mixture was incubated at 25 °C for 30 min in a 96-well black microplate (Nunc A/S, Thermo Fisher Scientific Inc., Waltham, Massachusetts, USA). Subsequently, the fluorescence was measured using a multimode plate reader (BioTek Cytation™ 3, Winooski, Vermont, USA) with excitation and emission wavelengths set at 485 nm and 520 nm, respectively. The obtained data were normalized to the total protein content and expressed relative to the control (100%). Total protein was quantified using the Bradford assay (Bio-Rad Protein Assay, Bio-Rad Laboratories, Hercules, California, USA) (Bradford, 1976). All experiments were performed in triplicate.

### Malondialdehyde levels

The amount of Malondialdehyde (MDA) was quantified using a colorimetric method based on the reaction between MDA and thiobarbituric acid. A lipid peroxidation (MDA) assay kit from Sigma-Aldrich was employed for the measurement of MDA activity. In brief, the samples (approximately 4000 individuals) were homogenized on ice using 200 μL of MDA lysis buffer and sonication. The resulting homogenate was then centrifuged at 13,000×*g* for 10 min at 4 °C, and the supernatant was collected in new tubes for subsequent analysis, following the manufacturer's instructions. The absorbance of the samples was measured at an optical density of 532 nm.

### Acetylcholinesterase activity

Acetylcholinesterase (AChE) participates in the hydrolysis of acetylthiocholine, leading to the production of thiocholine. The activity of AChE was determined using a Colorimetric AChE assay kit obtained from Abcam (Cambridge, UK). Briefly, approximately 4000 rotifers were homogenized in 300 μL of precooled phosphate buffer (0.1 M, pH 8.0) using sonication, following the method described in previous studies^62,68^. The resulting homogenate was then centrifuged at 3000×*g* for 30 min at 4 °C, and the supernatant was transferred to new tubes for subsequent analysis, as per the manufacturer's instructions. The activity of the enzyme was measured using 5,5-dithiobis (2-nitrobenzoic acid) at an absorbance of 410 nm.

### Superoxide dismutase activity

Superoxide dismutase (SOD) is responsible for catalyzing the dismutation of the superoxide anion into hydrogen peroxide (H_2_O_2_), which can further react with WST-1 (2-[4-iodophenyl]-3-[4-nitrophenyl]-5-[2,4-disulfophenyl]-2H-tetrazolium, monosodium salt) to generate a visible formazan dye. The quantity of formazan dye produced is inversely proportional to the SOD activity; higher SOD activity leads to lower formazan synthesis. The measurement of SOD activity was conducted using a colorimetric SOD activity assay kit (Abcam, Cambridge, UK) following the provided manufacturer's instructions. To perform the assay, approximately 4000 rotifers were homogenized on ice using 150 μL of 0.1 M Tris/HCl buffer (pH 7.4) containing 0.5% Triton X-100, 5 mM β-mercaptoethanol, and 0.1 mg/mL PMSF through sonication. The resulting homogenate was centrifuged at 14,000×*g* for 5 min at 4 °C, and the supernatant was transferred to a clean tube for further analysis according to the manufacturer's instructions. The activity of SOD was measured by monitoring the absorbance at 440 nm.

### Catalase activity

Catalase (CAT) activity was assessed through a CAT-H_2_O_2_-probe reaction, where CAT in the samples reacted with H_2_O_2_ to convert H_2_O and O_2_. Any remaining unreacted H_2_O_2_ then reacted with the probe, producing a colorimetric signal. The measurement of CAT activity was performed using a CAT activity assay kit obtained from Abcam (Cambridge, UK), following the provided manufacturer's instructions. To conduct the assay, approximately 4000 rotifers were homogenized using 200 μL of ice-cold assay buffer through sonication. The resulting homogenate was centrifuged at 10,000×*g* for 15 min at 4 °C, and the supernatant was carefully transferred to a new tube for subsequent analysis, following the manufacturer's instructions. CAT activity was determined by measuring the absorbance at 570 nm.

The measurement of enzyme activities was conducted using a commercially available enzyme kit, following standard laboratory protocols. Colorimetric signals were detected using a multimode plate reader (BioTek Cytation™ 3, Winooski, VT, USA) at the appropriate wavelengths for each enzyme. To account for variations in sample protein content, all enzyme activities were normalized to total protein and expressed relative to the control group (set as 100%). The quantification of total protein was carried out using the Bradford assay^69^. All experiments were performed in triplicate to ensure reproducibility and accuracy of the results (Supplementary Fig. S1).

### Statistical analysis

Statistical analysis was conducted on the measured values of various rotifer population growth parameters, including population growth rate (*r*), mixis rate, and fertilization rate, as well as the relative enzymatic/biomarker activities such as relative gene expression, ROS levels, MDA level, AChE, SOD, and CAT activities in the experimental rotifer. To investigate the relationship between MPs and the growth of the rotifer population, an independent t-test (n = 3) was performed. This test compared the growth parameters of the experimental rotifer in the presence and absence of MPs to identify any significant differences. Furthermore, to assess the effect of MPs on the relative enzymatic/biomarker activities, including relative gene expression, ROS levels, MDA level, AChE, SOD, and CAT activities, a one-way ANOVA was conducted (n = 3). Prior to the ANOVA, Levene's test was used to confirm the homogeneity of variances. For post-hoc analysis, the Tukey HSD test was employed when the *p*-value was found to be less than 0.05. The statistical program SPSS (Sigma Stat 3.0, SPSS, Chicago, U.S.A) was utilized for all the statistical analyses.

### Ethics statement

In conducting our experiment, we are committed to upholding the highest ethical standards. We will ensure the welfare of experimental rotifer by providing appropriate environmental conditions and minimizing harm, thus avoiding unnecessary distress or suffering. Transparency and informed consent will be upheld throughout the experiment to maintain ethical integrity and adhere to responsible research practices.

### Supplementary Information

Supplementary Figure S1.

## Data Availability

The data that support the findings of this study are available from the corresponding author, T. Seong, upon reasonable request.
