# Author Correction: Effects of microplastics on reproductive characteristics and mechanisms of the marine rotifer *Brachionus plicatilis*

**DOI:** 10.1038/s41598-024-69472-7

**Published:** 2024-08-07

**Authors:** Taekyoung Seong, Sae Yamamoto, Hisayuki Nakatani, Mitsuharu Yagi, Yusaku Kyozuka, Glenn Satuito, Hee‑Jin Kim

**Affiliations:** 1 Graduate School of Integrated Science and Technology, Nagasaki University, 1‑14 Bunkyo, Nagasaki, 852‑8521 Japan; 2 Co‑Creation Management Department, Ryukyu University, 1 Chihara, Nishihara‑Cho, Nakagami‑Gun, Okinawa Prefecture 903‑0213 Japan; 3 Faculty of Fisheries, Nagasaki University, 1‑14 Bunkyo, Nagasaki, 852‑8521 Japan; 4 Polymeri Materials Laboratory, Chemistry and Materials Program, Nagasaki University, 1‑14 Bunkyo, Nagasaki, 852‑8521 Japan; 5 Organization for Marine Science and Technology, Nagasaki University, 1‑14 Bunkyo, Nagasaki, 852‑8521 Japan

Correction to: *Scientific Report* 10.1038/s41598-024-65047-8, publsihed online 02 July 2024

The original version of this Article contained errors in the Materials and methods section, under the subheading ‘Relative gene expression’.

As a result,

“Reverse transcription quantitative polymerase chain reaction (RT-qPCR) was performed using 1 μL of cDNA template, 0.5 μL of forward and reverse primers (10 μM), and 10 μL of TB Green Premix Ex Taq (2 ×) (Takara Bio Inc, Shiga, Japan), making a total volume of 20 μL.”

now reads:

“Reverse transcription quantitative polymerase chain reaction (RT-qPCR) was performed using 1 μg of cDNA template, 0.5 μL of forward and reverse primers (100 μM), and 10 μL of TB Green Premix Ex Taq (2 ×) (Takara Bio Inc, Shiga, Japan), making a total volume of 20 μL.”

The original Article has been corrected.

