# Supplementary material for: Effects of microplastics on reproductive characteristics and mechanisms of the marine rotifer Brachionus plicatilis
Source: Sci Rep. 2024 Jul 2;14:15213. doi: 10.1038/s41598-024-65047-8 (PMC11219915; doi:10.1038/s41598-024-65047-8)

**Supplementary Information**

**Effects of microplastics on reproductive characteristics and mechanisms of the marine rotifer *Brachionus plicatilis***

Taekyoung Seong ^a,d^, Sae Yamamoto ^b^, Hisayuki Nakatani ^a,c^, Mitsuharu Yagi ^a,b^, Yusaku Kyozuka ^a^, Glenn Satuito ^a,b^, and Hee-Jin Kim ^a,b*^

^a^ Organization for Marine Science and Technology, Nagasaki University, 1-14 Bunkyo, Nagasaki 852-8521, Japan

^b^ Faculty of Fisheries, Nagasaki University, 1-14 Bunkyo, Nagasaki 852-8521, Japan

^c^ Polymeri Materials Laboratory, Chemistry and Materials Program, Nagasaki University, 1-14 Bunkyo, Nagasaki 852-8521, Japan

^d^ Co-Creation Management Department, Ryukyu University, 1 Chihara, Nishihara-cho, Nakagami-gun, Okinawa Prefecture, 903-0213, Japan

* Corresponding author.

*E-mail address*: heejin@nagasaki-u.ac.jp (H.-J. Kim)

Figure S1. Fluorescent observations of experimental rotifers exposed to fluorescent microbeads (3.00 µm, 1.0 µg/mL, Fluoresbrite YG Microspheres, PolyScience, Illinois, USA, Ex 441 nm, Em 486 nm) over a three-day period. (a) Experimental rotifer after one day of exposure to fluorescent microbeads. (b) Experimental rotifer after two days of exposure to fluorescent microbeads. (c) Experimental rotifer after three days of exposure to fluorescent microbeads.


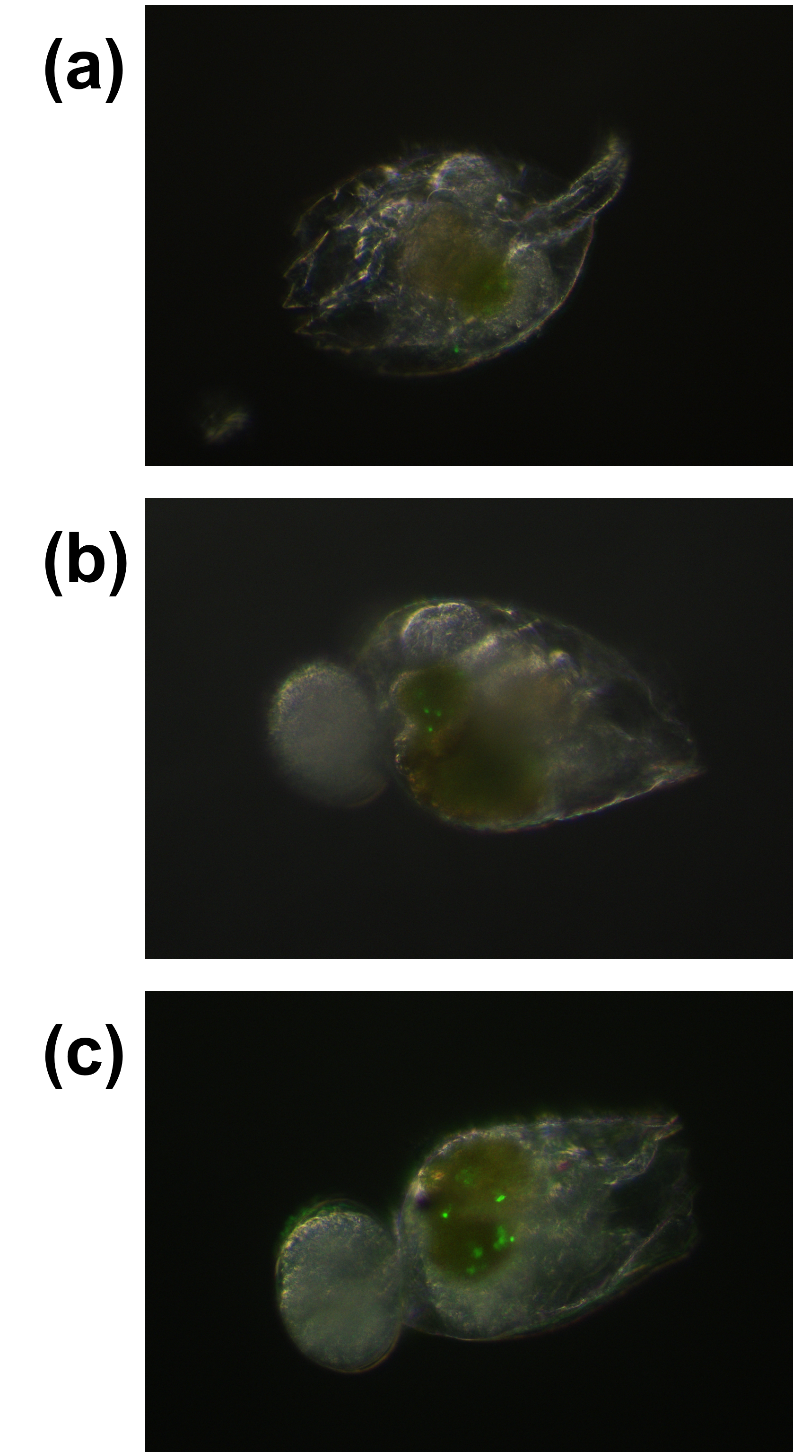

Supplement: Supplementary file 1 — Supplementary Figure S1. [file 41598_2024_65047_MOESM1_ESM.docx]
